# Prevalence and Determinants of Blood Exposure Accidents Among Health Care Workers in Kalemie, Democratic Republic of the Congo: Cross-Sectional Study

**DOI:** 10.2196/97009

**Published:** 2026-08-05

**Authors:** Joe Kabamba Dibwe, Fiston Ilunga Mbayo, Papy Mukalay Muvumbu, Gloire Kasongo Lupitshi, Jean Claude Banze Kabwe Ilunga, Didier Sashila Mukebo, Dieudonné Mukendi Mpunga

**Affiliations:** 1 Higher Institute of Medical Techniques of Kongolo Kongolo, Tanganyika the Democratic Republic of the Congo; 2 Provincial Health Division of Tanganyika Kalemie, Tanganyika the Democratic Republic of the Congo; 3 School of Public Health University of Malemba Nkulu Malemba Nkulu, Haut Lomami the Democratic Republic of the Congo; 4 Public Health Malemba Nkulu General Reference Hospital Malemba Nkulu, Haut Lomami the Democratic Republic of the Congo; 5 Higher Institute of Medical Techniques of Manono Manono, Tanganyika the Democratic Republic of the Congo; 6 University of Kamina Kamina, Haut-Lomami the Democratic Republic of the Congo; 7 National Malaria Control Program Democratic Republic of Congo Kinshasa, Kinshasa the Democratic Republic of the Congo; 8 School of Public Health Medicine University of Kinshasa Kinshasa, Kinshasa the Democratic Republic of the Congo

**Keywords:** blood exposure accidents, occupational hazards, health care workers, infection prevention and control, standard precautions, personal protective equipment, Democratic Republic of the Congo, sub-Saharan Africa

## Abstract

**Background:**

Blood exposure accidents (BEAs) remain a major occupational hazard for health care providers, particularly in low‑resource settings. Despite their preventable nature, BEAs continue to occur frequently due to gaps in compliance with standard precautions and limited institutional surveillance.

**Objective:**

This study aimed to determine the prevalence and factors associated with BEAs among health care providers in Kalemie, Tanganyika Province, Democratic Republic of the Congo.

**Methods:**

A cross-sectional study was conducted in March 2024 among 316 health care providers randomly selected from 34 health facilities: public facilities (20/34, 58.8%), private facilities (9/34, 26.5%), and faith‑based facilities (5/34, 14.7%). Data were collected using a structured questionnaire adapted from World Health Organization and Centers for Disease Control and Prevention tools. Logistic regression was performed to identify predictors of BEAs, with adjusted odds ratios (AORs), 95% CIs, and *P* values reported.

**Results:**

The lifetime prevalence of BEAs was 65.8% (208/316), and the 12-month prevalence was 60.4% (191/316). The most frequent causes were unexpected patient movement (81/191, 42.4%) and lack of attention (66/191, 34.6%). Needlestick injuries accounted for 59.2% (113/191) of exposures. Only 36.1% (69/191) of incidents were formally reported. Multivariate analysis identified two independent predictors: (1) noncompliance with standard precautions (AOR 2.921, 95% CI 1.503-5.677; *P*=.001) and (2) facility type (working in secondary‑level facilities was protective; AOR 0.310, 95% CI 0.144-0.670; *P*=.003).

**Conclusions:**

BEAs were highly prevalent among health care providers in Kalemie. Most incidents were preventable, and noncompliance with standard precautions significantly increased risk. Strengthening infection prevention and control measures, ensuring consistent availability of personal protective equipment, and establishing effective surveillance systems are urgently needed to protect health care workers and reinforce health system resilience.

## Introduction

Occupational exposure to blood and infectious body fluids remains a major public health concern, particularly for health care workers in low‑resource settings. Blood exposure accidents (BEAs) are defined as any accidental percutaneous or mucocutaneous contact with blood or biological fluids potentially contaminated with pathogens such as bacteria, viruses, parasites, or fungi [1,2]. These exposures typically occur through needlestick injuries, cuts, or splashes during clinical procedures. Beyond blood, other biological fluids—including cerebrospinal, synovial, amniotic, and genital secretions—may also transmit infections even when not visibly contaminated [3]. According to the World Health Organization (WHO), approximately 3 million of the 35 million health care workers worldwide are exposed to bloodborne pathogens annually [4,5]. Recent global evidence confirms the persistence of this risk: a meta‑analysis published in 2022 reported a lifetime prevalence of occupational blood exposure of 56.6% and a 12‑month prevalence of 39% among health care workers worldwide [6]. More recent studies highlight organizational and systemic determinants: a longitudinal cohort in France identified irregular schedules and reliance on external staff as predictors of BEAs [7], whereas a systematic review in 2023 found a pooled global prevalence of needlestick injuries of 40.9% among nurses, with higher rates in low- and middle-income countries [8]. In addition, the WHO reported in 2023 that 41% of health workers in Africa experience at least one percutaneous injury annually, underscoring the magnitude of the problem [9]. The risk of transmission varies by pathogen: 10% to 40% for hepatitis B virus, approximately 2% for hepatitis C virus, and approximately 0.3% for HIV following a needlestick injury [10,11]. The WHO estimates that nearly 2 million needlestick injuries occur annually among health care workers, with 40% to 60% of hepatitis B and hepatitis C virus infections in this population being occupationally acquired [12]. Recent studies confirm the persistence of BEAs: prevalence rates have reached 93% in Cameroon [13], 78.9% among nursing students in Morocco [14], and 31% in India [15]. In Ethiopia and Nigeria, lifetime prevalence exceeds 60% [16,17]. In the Democratic Republic of the Congo (DRC), the problem is particularly acute. A study in Lubumbashi reported a prevalence of 73.2% among health care workers [18,19]. Noncompliance with standard precautions was identified as the strongest predictor (adjusted odds ratio [AOR] 2.921, 95% CI 1.503-5.677), confirming that most BEAs could be prevented through simple measures such as consistent use of personal protective equipment (PPE) and avoidance of needle recapping [19]. These findings highlight systemic gaps in infection prevention and control across provinces in the DRC. Multiple factors contribute to BEAs, including insufficient infection prevention training, inadequate PPE, poor compliance with standard precautions, absence of infection control committees, and overcrowded or poorly designed workspaces [20,21]. While high‑income countries have implemented surveillance systems and continuous training programs [22], many African countries still face underreporting and poor documentation of BEAs [23]. This study aimed to determine the factors associated with BEAs among health care providers in Kalemie, DRC, hypothesizing that noncompliance with standard precautions significantly increases the occurrence of BEAs.

## Methods

### Study Design and Setting

We conducted a cross-sectional study in March 2024 among health care providers in Kalemie, Tanganyika Province, eastern DRC. The city comprises 2 health zones (Kalemie and Nyemba) covering 11 health areas and a total of 45 public, private, and faith-based health facilities.

### Sample Size and Sampling Technique

The sample size was calculated using the single population proportion formula [24]: *n* = [(*z*α/2)^2^ × *p* (1 − *p*)]/*d*^2^, where *z*=1.96 (for 95% confidence), *p*=73.2% (estimated BEA prevalence from the aforementioned Lubumbashi study), and *d*=0.05 (margin of error). After accounting for a 10% nonresponse rate, the final sample size was 335.

A 2‑stage sampling approach was applied. Although clustering was considered, no formal adjustment for design effect was performed as the calculated sample size was deemed sufficient to ensure representativeness. The final number of respondents was 316, corresponding to a response rate of 94.3% (316/335); the difference was due to nonresponses.

### Recruitment Procedure

From the 45 health facilities listed in the National Health Information System (NHIS), 34 (75.6%) were randomly selected using a computer‑generated list, ensuring proportional representation of public, private, and faith‑based institutions. Within each selected facility, staff rosters were systematically validated by managers against official NHIS records to guarantee accuracy and completeness. The number of participants per facility was determined proportionally to staff size, and providers were then chosen using a random number generator. This systematic validation of lists and proportional allocation ensured representativeness and minimized selection bias.

### Inclusion and Exclusion Criteria

Inclusion criteria were nurses, physicians, midwives, and laboratory technicians with direct contact with blood or body fluids. Exclusion criteria were providers with less than 1 month of professional experience to reduce onboarding variability.

### Data Collection Tools and Procedures

Data were collected using a structured questionnaire adapted from validated WHO and Centers for Disease Control and Prevention (CDC) instruments [25-27]. The tool comprised closed‑ended items covering sociodemographic characteristics, knowledge of BEAs, attitudes toward reporting and prevention, compliance with standard precautions, and history of occupational exposure. Prior to implementation, the questionnaire was pilot‑tested at Baraka Health Post and Shalom Polyclinic to ensure clarity and contextual relevance. Seven data collectors and 1 supervisor received 2 days of standardized training focused on interview techniques, ethical considerations, and data quality assurance. Reliability testing yielded a Cronbach α coefficient of 0.766, indicating acceptable internal consistency. Data collection was conducted through face-to-face interviews using the KoboCollect (Kobo) software installed on tablets. Each interviewer completed approximately 10 provider interviews per day over a 5-day period. Supervisory checks were performed daily to validate the completeness and accuracy of entries. The use of electronic data capture minimized transcription errors and ensured secure storage of anonymized records.

### Variables

The dependent variable was occupational exposure to blood within the previous 12 months, defined as any percutaneous or mucocutaneous contact with potentially infectious biological fluids. Independent variables included the type of health facility, categorized as secondary level (general referral hospitals, referral health centers, and polyclinics) vs primary level (health centers, health posts, and dispensaries). Compliance with standard precautions was dichotomized as “always” vs “not always.” Needle recapping was assessed through self‑reported frequency and classified as “never” vs “ever.” Availability and use of PPE were measured on an ordinal scale (“rarely,” “usually,” and “always”). Syringe reuse was recorded as a binary variable (“yes” or “no”). Sociodemographic characteristics were also collected, including age (categorized), sex, marital status, professional category, years of professional experience, and daily working hours.

### Statistical Analysis

Data were exported from KoboCollect to Microsoft Excel 2016 and analyzed using SPSS (version 25.0; IBM Corp). Descriptive statistics were computed, including frequencies, percentages, medians, and IQRs. Normality of continuous variables was assessed using the Kolmogorov-Smirnov test [28]. Knowledge scores were derived from 31 items and categorized as optimal (>85%), moderate (70%-85%), or poor (<70%). Bivariate analyses were performed using chi‑square tests, and variables with a *P* value of .20 or lower were retained for multivariate modeling. Logistic regression was conducted to identify independent predictors of BEAs, with AORs and 95% CIs reported. Both crude (odds ratio) and adjusted (AOR) estimates were presented, and potential confounding factors were explicitly discussed. Model diagnostics included assessment of multicollinearity using the variance inflation factor (<2), evaluation of model fit using the Hosmer-Lemeshow goodness‑of‑fit test, and discrimination using the area under the receiver operating characteristic curve [29]. Statistical significance was set at a *P* value below .05.

### Ethical Considerations

This study was conducted and reported in accordance with the STROBE (Strengthening the Reporting of Observational Studies in Epidemiology) guidelines [30]. Ethics approval was obtained from the ethics committee of the School of Public Health, University of Kinshasa (reference ESP/CE/38/2024). Written informed consent was obtained from all participants prior to their inclusion. Confidentiality was safeguarded by anonymizing the data and storing them securely, accessible only to authorized researchers. No financial compensation was provided to participants for their involvement in the study.

## Results

### Sociodemographic and Professional Characteristics

A total of 316 health care providers participated (response rate: 316/335, 94.3%). Most (n=256, 81%) worked in secondary‑level facilities (general referral hospitals, referral health centers, and polyclinics). Most were female (n=179, 56.6%), married (n=230, 72.8%), and aged 40 years or older (n=128, 40.5%). Nurses represented 78.2% (n=247) of the sample. Median professional experience was 9 (IQR 5‑15) years, and median workload was 8 (IQR 7‑9) hours per day, as shown in Table 1.

**Table 1:** Sociodemographic and professional characteristics of health care providers surveyed in Kalemie, Tanganyika Province, Democratic Republic of the Congo, in March 2024 (cross‑sectional study; N=316).

Characteristic and category			Values
Type of health facility, n (%)			
	General referral hospital, referral health center, or polyclinic	256 (81)	
	Health center, health post, or dispensary	60 (19)	
Age (y), median (IQR)			38 (30‑46)
	19-29, n (%)	66 (20.9)	
	30-39, n (%)	122 (38.6)	
	≥40, n (%)	128 (40.5)	
Sex, n (%)			
	Male	137 (43.4)	
	Female	179 (56.6)	
Marital status, n (%)			
	Single	57 (18)	
	Married	230 (72.8)	
	Common-law union	4 (1.3)	
	Widowed	25 (7.9)	
Professional category, n (%)			
	Nurse	247 (78.2)	
	Midwife or birth attendant	18 (5.7)	
	Laboratory technician	21 (6.6)	
	Physician	30 (9.5)	
Department of assignment, n (%)			
	Surgery or operating room	31 (9.8)	
	Obstetrics and gynecology	61 (19.3)	
	Laboratory	31 (9.8)	
	Internal medicine	40 (12.7)	
	Pediatrics	26 (8.2)	
	General services	117 (37)	
	Emergency	10 (3.2)	
Experience (y), median (IQR)			9 (5‑15)
	<5, n (%)	67 (21.2)	
	≥5, n (%)	249 (78.8)	
Workload (h), median (IQR)			8 (7‑9)
	≤8, n (%)	209 (66.1)	
	>8, n (%)	107 (33.9)	

### Prevalence and Circumstances of BEAs

Overall, 65.8% (208/316) reported at least one BEA during their career, and 60.4% (191/316) reported one within the previous 12 months. The leading causes were unexpected patient movement (81/191, 42.4%) and lack of attention (66/191, 34.6%). Most incidents occurred in treatment rooms (64/191, 33.5%), delivery rooms (33/191, 17.3%), and operating rooms (27/191, 14.1%).

Needlestick injuries accounted for 59.2% (113/191) of exposures, followed by splashes on damaged skin (43/191, 22.5%) and on mucous membranes (18/191, 9.4%). Hollow needles (77/191, 40.3%) and defective surgical equipment (49/191, 25.7%) were the objects most frequently responsible, as shown in Table 2. Only 36.1% (69/191) of BEAs were formally reported; among the 122 non‑reported cases, the main reason was the perception that the incident was not risky (70/122, 57.4%).

**Table 2:** Prevalence and circumstances of blood exposure accidents (BEAs) among health care providers in Kalemie, Tanganyika Province, Democratic Republic of the Congo, in March 2024 (N=316).

Characteristic			Participants, n (%)
BEA occurrence during professional career			
	No	108 (34.2)	
	Yes	208 (65.8)	
BEA occurrence in the previous 12 mo			
	No	125 (39.6)	
	Yes	191 (60.4)	
Cause of BEA (n=191)			
	Unexpected patient movement	81 (42.4)	
	Lack of attention	66 (34.6)	
	Lack of safety devices	23 (12)	
	Work context (stress or overload)	10 (5.2)	
	Lack of practical experience	7 (3.7)	
	Restricted workspace	4 (2.1)	
Location of occurrence (n=191)			
	Treatment room	64 (33.5)	
	Delivery room	33 (17.3)	
	Operating room	27 (14.1)	
	Laboratory	21 (11)	
	Patient room	17 (8.9)	
	Emergency room	17 (8.9)	
	Other	8 (4.2)	
	Vaccination session	4 (2.1)	
Mechanism of occurrence (n=191)			
	Needlestick	113 (59.2)	
	Splash on damaged skin	43 (22.5)	
	Splash on mucous membrane	18 (9.4)	
	Blade injury	14 (7.3)	
	Other	3 (1.6)	
Object responsible (n=191)			
	Hollow needle (syringe)	77 (40.3)	
	Defective surgical equipment	49 (25.7)	
	Catheter	29 (15.2)	
	Suture needle	24 (12.6)	
	Blade	12 (6.3)	
Risky procedure responsible for BEA (n=191)			
	Intramuscular injection	62 (32.5)	
	Suturing	27 (14.1)	
	Needle insertion or removal	22 (11.5)	
	Blood sampling	21 (11)	
	Other	19 (9.9)	
	Surgical procedure	14 (7.3)	
	Delivery management	13 (6.8)	
	Puncture	7 (3.7)	
	Recapping used needle	6 (3.1)	

### Knowledge, Attitudes, and Practices

Nearly all participants (309/316, 97.8%) had heard of BEAs, mainly through formal education (125/316, 40.5%) and training (125/316, 40.5%). However, 72.8% (230/316) had never received specific training on standard precautions, and 94.3% (298/316) demonstrated poor knowledge levels.

Attitudes were mixed: 48.4% (153/316) strongly agreed on the importance of reporting BEAs, 72.2% (228/316) emphasized the importance of knowing the patient’s serological status, and 73.4% (232/316) expressed willingness to initiate postexposure prophylaxis.

In practice, 76.6% (242/316) claimed to always follow standard precautions, but only 25% (79/316) consistently washed their hands before and after patient contact. Needle recapping was reported by 27.8% (88/316), PPE was always used by 19.6% (62/316), and syringe reuse occurred in 16.5% (52/316) of cases, as shown in Table 3.

**Table 3:** Knowledge, attitudes, and practices regarding blood exposure accidents (BEAs) among health care providers in Kalemie, Tanganyika Province, Democratic Republic of the Congo, in March 2024 (N=316).

Characteristic		Participants, n (%)
Had information about BEAs		
	No	7 (2.2)
	Yes	309 (97.8)
Source of information (n=309)		
	School or university	125 (40.5)
	Radio	5 (1.6)
	Colleague	25 (8.1)
	Training	125 (40.5)
	Official memo	8 (2.5)
	Other sources	21 (6.8)
Training on standard precautions		
	No	230 (72.8)
	Yes	86 (27.2)
Level of knowledge on BEAs		
	Low	298 (94.3)
	Moderate	16 (5.1)
	Optimal	2 (0.6)
Importance of reporting BEAs		
	Strongly disagree	26 (8.2)
	Slightly agree	29 (9.2)
	Agree	106 (33.5)
	Strongly agree	153 (48.4)
	Do not know	2 (0.6)
Importance of knowing patients’ serostatus		
	Strongly disagree	10 (3.2)
	Slightly agree	9 (2.8)
	Agree	67 (21.2)
	Strongly agree	228 (72.2)
	Do not know	2 (0.6)
Importance of knowing one’s own serostatus		
	Strongly disagree	6 (1.9)
	Slightly agree	10 (3.2)
	Agree	81 (25.6)
	Strongly agree	218 (69)
	Do not know	1 (0.3)
Importance of PPE^a^ use for BEA prevention		
	Strongly disagree	28 (8.9)
	Slightly agree	69 (21.8)
	Agree	139 (44)
	Strongly agree	80 (25.3)
Postexposure prophylaxis consideration		
	No	84 (26.6)
	Yes	232 (73.4)
Compliance with standard precautions		
	Never	1 (0.3)
	Rarely	36 (11.4)
	Usually	37 (11.7)
	Always	242 (76.6)
Hand hygiene		
	Never	14 (4.4)
	Rarely	67 (21.2)
	Usually	156 (49.4)
	Always	79 (25)
Needle recapping		
	Never	89 (28.2)
	Rarely	49 (15.5)
	Usually	90 (28.5)
	Always	88 (27.8)
Mouth pipetting		
	Never	182 (57.6)
	Rarely	7 (2.2)
	Usually	11 (3.5)
	Not applicable	116 (36.7)
Availability of PPE		
	Rarely	99 (31.3)
	Usually	137 (43.4)
	Always	80 (25.3)
Use of PPE		
	Never	1 (0.3)
	Rarely	92 (29.1)
	Usually	161 (50.9)
	Always	62 (19.6)
Reuse of syringes		
	No	264 (83.5)
	Yes	52 (16.5)

^a^PPE: personal protective equipment.

### Factors Associated With BEAs

Bivariate analysis identified 6 factors associated with BEAs, with exact *P* values: facility type (*P*<.001), daily working hours (*P*=.001), compliance with standard precautions (*P*<.001), needle recapping (*P*<.001), PPE availability (*P*=.03), and PPE use (*P*=.003).

Multivariate logistic regression confirmed two independent predictors: (1) noncompliance with standard precautions (AOR 2.921, 95% CI 1.503‑5.677; *P*=.001) and (2) facility type (AOR 0.310, 95% CI 0.144‑0.670; *P*=.003), as shown in Table 4.

**Table 4:** Bivariate and multivariate analysis of factors associated with blood exposure accidents (BEAs) among health care providers in Kalemie, Tanganyika Province, Democratic Republic of the Congo, in March 2024 (N=316).

Characteristic			BEA occurrence, n (%)					Bivariate analysis					Multivariate analysis		
			No (n=125)		Yes (n=191)		OR^a^ (95% CI)			*P* value		Adjusted OR (95% CI)			*P* value
Type of health facility															
	General referral hospital, referral health center, or polyclinic	88 (70.4)		168 (88.0)		3.071 (1.718-5.490)			*<.001* ^b^		0.310 (0.144-0.670)			*.003* ^b^	
	Health center, health post, or dispensary	37 (29.6)		23 (12.0)		1 (reference)			—^c^		1 (reference)			—	
Sex															
	Male	58 (46.4)		79 (41.4)		0.815 (0.517-1.284)			.38		—			—	
	Female	67 (53.6)		112 (58.6)		1 (reference)			—		—			—	
Age group (y)															
	19-29	32 (25.6)		34 (17.8)		0.681 (0.374-1.240)			.21		—			—	
	30-39	43 (34.4)		79 (41.4)		1.178 (0.704-1.969)			.53		—			—	
	≥40	50 (40)		78 (40.8)		1 (reference)			—		—			—	
Professional category															
	Midwife	7 (5.6)		11 (5.8)		1.179 (0.327-4.250)			.80		—			—	
	Nurse	101 (80.8)		146 (76.4)		1.084 (0.440-2.669)			.86		—			—	
	Physician	8 (6.4)		22 (11.5)		2.062 (0.631-6.739)			.23		—			—	
	Laboratory technician	9 (7.2)		12 (6.3)		1 (reference)			—		—			—	
Department of assignment															
	Surgery or operating room	6 (4.8)		25 (13.1)		2.206 (0.663-7.344)			.20		0.458 (0.130-1.613)			.22	
	Obstetrics and gynecology	20 (16)		41 (21.5)		1.085 (0.412-2.859)			.87		0.521 (0.171-1.589)			.25	
	Laboratory	15 (12)		16 (8.4)		0.565 (0.193-1.649)			.30		1.735 (0.314-9.593)			.53	
	Internal medicine	18 (14.4)		22 (11.5)		0.647 (0.233-1.795)			.40		1.352 (0.463-3.944)			.58	
	General services	55 (44)		61 (31.9)		0.577 (0.238-1.398)			.22		0.988 (0.370-2.641)			.98	
	Emergency	1 (0.8)		9 (4.7)		4.765 (0.518-43.798)			.17		0.282 (0.030-2.680)			.27	
	Pediatrics	9 (7.2)		17 (8.9)		1 (reference)			—		1 (reference)			—	
Experience (y)															
	<5	29 (23.2)		38 (19.9)		0.822 (0.476-1.420)			.48		—			—	
	≥5	96 (76.8)		153 (80.1)		1 (reference)			—		—			—	
Workload (h)															
	>8	56 (44.8)		51 (26.7)		2.228 (1.383-3.588)			*.001* ^b^		0.627 (0.359-1.093)			.10	
	≤8	69 (55.2)		140 (73.3)		1 (reference)			—		1 (reference)			—	
Training on standard precautions															
	No	95 (76)		135 (70.7)		0.761 (0.455-1.274)			.30		—			—	
	Yes	30 (24)		56 (29.3)		1 (reference)			—		—			—	
Compliance with standard precautions															
	Not always	14 (11.2)		38 (19.9)		2.915 (1.707-4.980)			*<.001* ^d^		2.921 (1.503-5.677)			*.001* ^b^	
	Always	111 (88.8)		153 (80.1)		1 (reference)			—		1 (reference)			—	
Needle recapping															
	Always, usually, or rarely	75 (60)		39 (20.4)		2.598 (1.573-4.292)			*<.001* ^d^		0.884 (0.459-1.703)			.71	
	Never	50 (40)		152 (79.6)		1 (reference)			—		1 (reference)			—	
Availability of PPE^e^															
	Not always	85 (68)		151 (79.1)		0.563 (0.337-0.940)			*.03* ^b^		0.760 (0.397-1.458)			.41	
	Always	40 (32)		40 (20.9)		1 (reference)			—		1 (reference)			—	
Use of PPE during procedures															
	Not always	39 (31.2)		54 (20.3)		2.362 (1.344-4.152)			*.003* ^b^		0.546 (0.263-1.135)			.11	
	Always	86 (68.8)		137 (71.7)		1 (reference)			—		1 (reference)			—	

^a^OR: odds ratio.

^b^*P*<.05.

^c^Not applicable.

^d^*P*<.001.

^e^PPE: personal protective equipment.

## Discussion

### Summary of Key Findings

This study highlights that BEAs remain a major occupational hazard among health care providers in Kalemie, Tanganyika Province, DRC. The strongest predictor was noncompliance with standard precautions [15], whereas working in secondary‑level facilities appeared protective.

### Interpretation and Comparison With Previous Studies

Our findings are consistent with those of recent African studies reporting high BEA prevalence: 93% in Cameroon [10], 78.9% in Morocco [11], more than 60% in Ethiopia [13,17], and more than 60% in Nigeria [14]. The Kalemie results reinforce the persistence of occupational risks across sub‑Saharan Africa.

The protective effect of secondary‑level facilities may be explained by better infrastructure, availability of PPE, and more structured infection prevention programs, as observed in Ethiopia [17] and Benin [18]. Conversely, primary‑level facilities often lack resources, increasing exposure risks [20].

The association between noncompliance with standard precautions and BEAs has been consistently documented [27]. In Kalemie, this factor nearly tripled the risk, confirming that most accidents are preventable through adherence to basic infection control measures. The apparent contradiction between bivariate and multivariate analyses regarding facility type likely reflects confounding factors: secondary facilities host more complex procedures and higher patient volumes, increasing crude risk, but once adjusted for PPE availability, training, and compliance, their structured infection control systems reduced overall risk. Similar methodological challenges have been highlighted in recent epidemiological modeling studies [31].

### Strengths and Limitations

The methodological rigor of this study, including the use of a validated questionnaire adapted from WHO and CDC tools [22,26], random sampling, and robust statistical diagnostics such as the Hosmer-Lemeshow test and the area under the receiver operating characteristic curve [28,29], supports the representativeness of the findings. Nevertheless, limitations must be acknowledged. Reliance on self‑reported data may have introduced social desirability bias, as suggested by the discrepancy between the high proportion of providers claiming to “always” follow standard precautions (242/316, 76.6%) and the finding that noncompliance was the strongest predictor. Recall bias may also have affected lifetime prevalence estimates. Measurement limitations exist because exposure was assessed exclusively through self‑report without external validation, and the cross‑sectional design precludes causal inference. Finally, generalizability is limited as this study was conducted in Kalemie and may not fully represent health care providers in other provinces of the DRC.

### Broader Implications

BEAs remain highly prevalent among health care providers in Kalemie. Most incidents are preventable through adherence to standard precautions, adequate provision of PPE, and establishment of surveillance systems. Strengthening institutional policies and continuous training are essential to reduce occupational risks and safeguard health care workers.

Beyond Kalemie, these findings highlight systemic gaps in infection prevention and control across sub‑Saharan Africa. Addressing these gaps requires coordinated action: investment in PPE, integration of infection control committees, and reinforcement of reporting and monitoring systems. At the global level, the persistence of BEAs underscores the need for harmonized occupational health policies and stronger advocacy for health care worker safety.

### Conclusions

BEAs remain a major occupational hazard for health care providers in Kalemie, DRC. With nearly two-thirds of providers reporting lifetime exposure and more than half exposed within the past year, the burden is substantial. Most incidents were preventable, and noncompliance with standard precautions emerged as the strongest predictor. Conversely, working in secondary‑level facilities appeared protective once confounding factors were accounted for.

These findings underscore the urgent need to strengthen infection prevention and control measures, ensure consistent availability and use of PPE, and establish effective surveillance and reporting systems. Beyond Kalemie, the results highlight systemic gaps across sub‑Saharan Africa, calling for coordinated national and international efforts to safeguard health care workers. Protecting those at the frontline is essential not only for their safety but also for the resilience of health systems.

## Data Availability

The datasets generated and analyzed during the current study are not publicly available due to confidentiality agreements with participating health facilities. However, anonymized data can be obtained from the corresponding author on reasonable request and with approval from the institutional ethics committee.

## Abbreviations

AOR: adjusted odds ratio
BEA: blood exposure accident
CDC: Centers for Disease Control and Prevention
DRC: Democratic Republic of the Congo
NHIS: National Health Information System
PPE: personal protective equipment
STROBE: Strengthening the Reporting of Observational Studies in Epidemiology
WHO: World Health Organization
